# The prevalence and clinical implication of rare germline deleterious alterations in Chinese patients with prostate cancer: A real‐world multicenter study

**DOI:** 10.1002/ctm2.527

**Published:** 2021-10-12

**Authors:** Xiaochen Fei, Liancheng Fan, Wei Chen, Wei Chen, Yiming Gong, Xinxing Du, Yanqing Wang, Yinjie Zhu, Jiahua Pan, Fangqin Wang, Wanbing Zhao, Tongtong Liu, Yining Yang, Baijun Dong, Wei Xue

**Affiliations:** ^1^ Department of Urology Renji Hospital School of Medicine Shanghai Jiao Tong University Shanghai China; ^2^ Department of Urology The First Affiliated Hospital of Wenzhou Medical University Zhejiang China; ^3^ Department of Urology Zhongshan Hospital Shanghai Medical College Fudan University Shanghai China; ^4^ GloriousMedTechnologyCo. Ltd Shanghai China

Dear Editor,

Germline deleterious alterations interrupting the function of DNA damage repair (DDR) have proven to be related to a high risk of prostate cancer (PCa), which are recommended for testing in general practice.^1^ Moreover, the genetic background has recently emerged as a potential factor in racial diversity, especially in the epidemiology of PCa.^2^ Since our understanding of the genomics was mostly derived from the Caucasian population, we conducted a real‐world multicenter retrospective study of 490 patients with PCa across distinct clinical states in order to better elucidate the prevalence and clinical implication of rare germline deleterious alterations in Chinese men.

A total of 490 patients with PCa, including 181 patients with localized PCa, 156 patients with metastatic hormone‐sensitive PCa, 147 patients with metastatic castration‐resistant PCa, and 6 patients with neuroendocrine‐differentiated PCa, were included in the present study (Figure 1A and Table 1). To explore the landscape of germline deleterious alterations, targeted gene sequencing of 50 genes covering DDR pathway genes and *HOXB13* was performed. In addition, concurrent *HSD3B1* genotypes were detected in 348 patients. Detailed sequencing and bioinformatics are summarized in Supplementary Methods.

**FIGURE 1:**
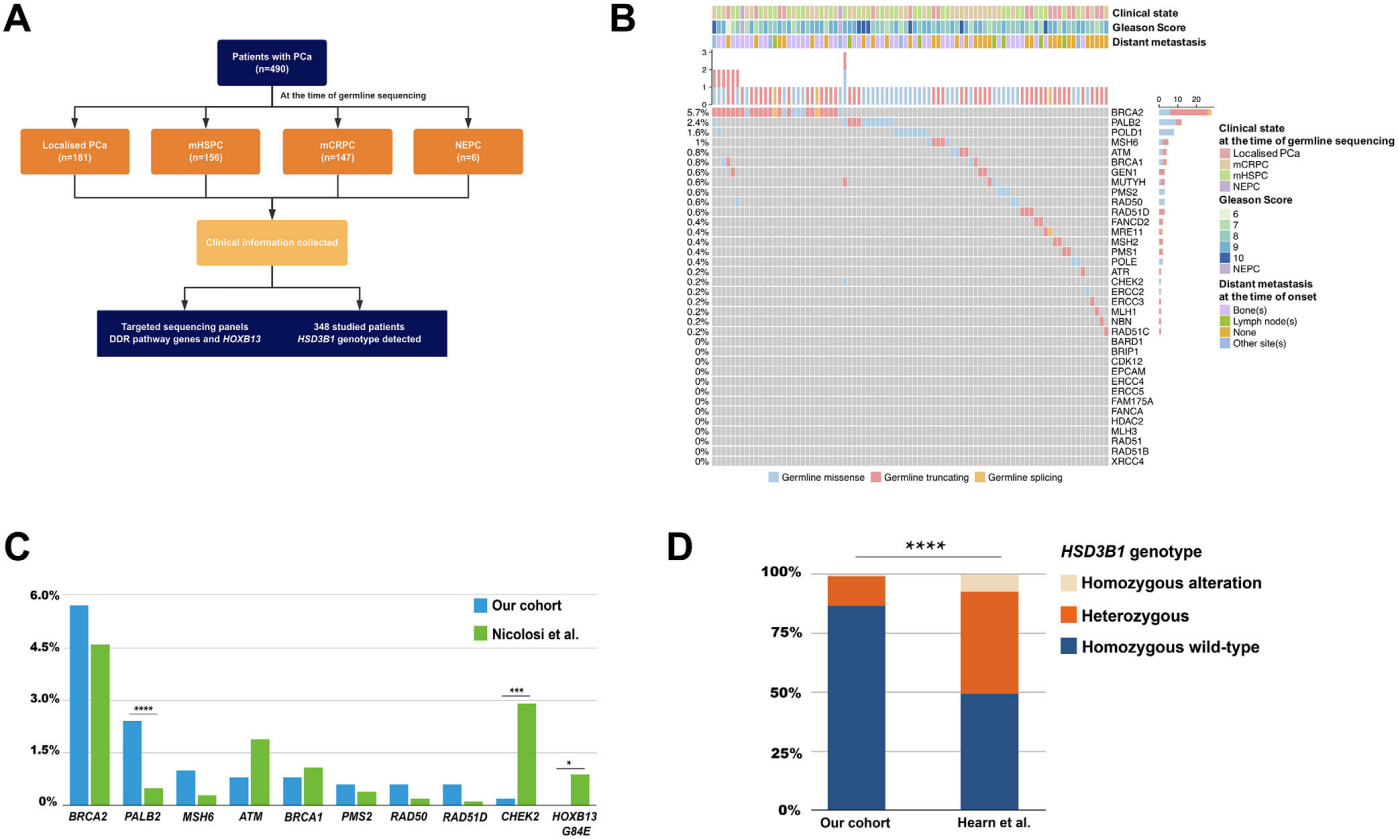
The landscape of germline deleterious alterations in DDR pathway genes and the comparison of rare germline deleterious alteration frequencies between our cohort and Caucasian's cohorts. (A) Overview of the studied patients. (B) Germline deleterious alterations of DDR pathway genes were identified in our cohort. Each column represents alteration detected in individual sample. Upper track shows total number of mutations. Frequencies of specific gene alterations are displayed on the right side. The color represents missense, truncating and splicing alterations. The upper horizonal display of the plot represents the clinical state at the time of germline sequencing, Gleason score, and the distant metastasis at the time of disease onset. (C) Bar plot shows the alteration frequency of DDR pathway genes and *HOXB13* between our cohort and the cohort by Nicolosi et al. study. (D) Bar plot shows the proportion of different genotypes of *HSD3B1* between our cohort and the cohort by Hearn et al. study. Fisher's exact test was used to compare the differences

**TABLE 1:** Clinical characteristics of the 490 studied patients

Clinical characteristics at onset time	Overall (*n* = 490)	gDDR altered (*n* = 81)	gDDR wild‐type (*n* = 409)	*p* value
Median age (IQR), year	67 (62‐72)	66 (61‐71)	68 (63‐72)	0.0830
PSA, *n* (%)				0.0075
0‐20 ng/ml	166 (33.9)	17 (20.1)	149 (36.3)	
20‐100 ng/ml	139 (28.4)	22 (27.2)	117 (28.7)	
> 100 mg/ml	185 (37.8)	42 (51.9)	143 (35.1)	
Gleason score, *n* (%)				0.0095
6	22 (4.5)	1 (1.2)	21 (5.1)	
7	137 (28.0)	12 (14.8)	125 (30.6)	
8	158 (32.2)	27 (33.3)	131 (32.0)	
9	136 (27.8)	32 (39.5)	104 (25.4)	
10	31 (6.3)	7 (8.6)	24 (5.9)	
Neuroendocrine	6 (1.2)	2 (2.5)	4 (0.98)	
Metastasis, *n* (%)				0.0149
Nonmetastasis	237 (48.4)	29 (35.8)	208 (50.9)	
With metastasis	253 (51.6)	52 (64.2)	201 (49.1)	
Lymph node	32	6	26	
Bone	215	44	171	
Visceral	6	2	4	
Family history of malignant tumors, *n* (%)	36 (7.3)	6 (7.4)	30 (7.3)	>0.9999

Although the distinctiveness in genetic background might play an essential role in the ethnic disparity in the same disease, the similar prevalence of germline deleterious alterations in DDR pathway genes between the Chinese and American populations has been reported a prior single‐center study.^3^ However, due to its relatively small sample size and limited detected genes, the ethnic differences in the germline genomes still remain to be further elucidated. To better determine the interracial heterogeneity in the genomics, we compared the incidence of germline DDR pathway gene alterations in our cohort with the unselected cohort by Nicolosi et al. study.^4^ In addition, the hereditary susceptibility along with *HOXB13* was included. Overall, 81 (16.5%) of the 490 studied patients with PCa harbored deleterious germline alterations in DDR pathway genes. The most frequently altered genes were *BRCA2* (5.7%, *n* = 28), followed by *PALB2* (2.4%, *n* = 12), *POLD1* (1.6%, *n* = 8), *MSH6* (1.0%, *n* = 5), and *ATM* (0.8%, *n* = 4) (Figure 1B). Interestingly, we found a relatively higher prevalence of germline deleterious alterations in *PALB2* (2.4% vs. 0.5%, *p *< 0.0001) and lower germline deleterious alteration rates in *CHEK2* (0.2% vs. 2.9%, *p *< 0.001) in our cohort in comparison with the cohort by Nicolosi et al. study (Figure 1C). Germline deleterious alterations in *PALB2*, interrupting the recombinational repair and the tumor suppression function, were associated with increased risk of various malignancies.^5^ However, the molecular pattern of germline *PALB2* alteration and its prognostic value need to be further elucidated. Similar to *PALB2*, *CHEK2* plays an important role in DDR and germline deleterious alterations in *CHEK2* may lead to the carcinogenesis of normal prostate cell.^6^ Further studies need to be conducted in a larger Chinese population to characterize possible alteration‐specific risks of *CHEK2* due to its low incidence.

Furthermore, a notable distinction of our cohort was the absence of HOXB13 p.G84E mutation compared to 0.9% in the cohort by Nicolosi et al. study (*p *= 0.02) (Figure 1C). *HOXB13* plays essential roles in prostate‐lineage differentiation and tumorigenesis, which is recommended for family counseling.^1^ Specially, the missense mutation G84E in the Caucasian populations has been identified to be strongly associated with increased PCa susceptibility, early onset, and aggressive disease.^7^ However, we failed to detect G84E in any of the 490 studied patients, instead, we found other four mutational sites, including G135E. The locations of germline deleterious alterations in the five most frequently altered genes are shown in Figure 2A. Since the recurrent mutation G135E was a founder mutation in a Chinese cohort,^8^ our results provided substantial support to the fact that HOXB13 p.G135E may be a prominent signature in the Chinese population. Although few PCa risk‐associated rare mutations in *HOXB13* have been identified to date, it is expected that additional mutations, such as G135E, will be found in ongoing studies in order to better understand the genetic mechanism underlying PCa.

**FIGURE 2:**
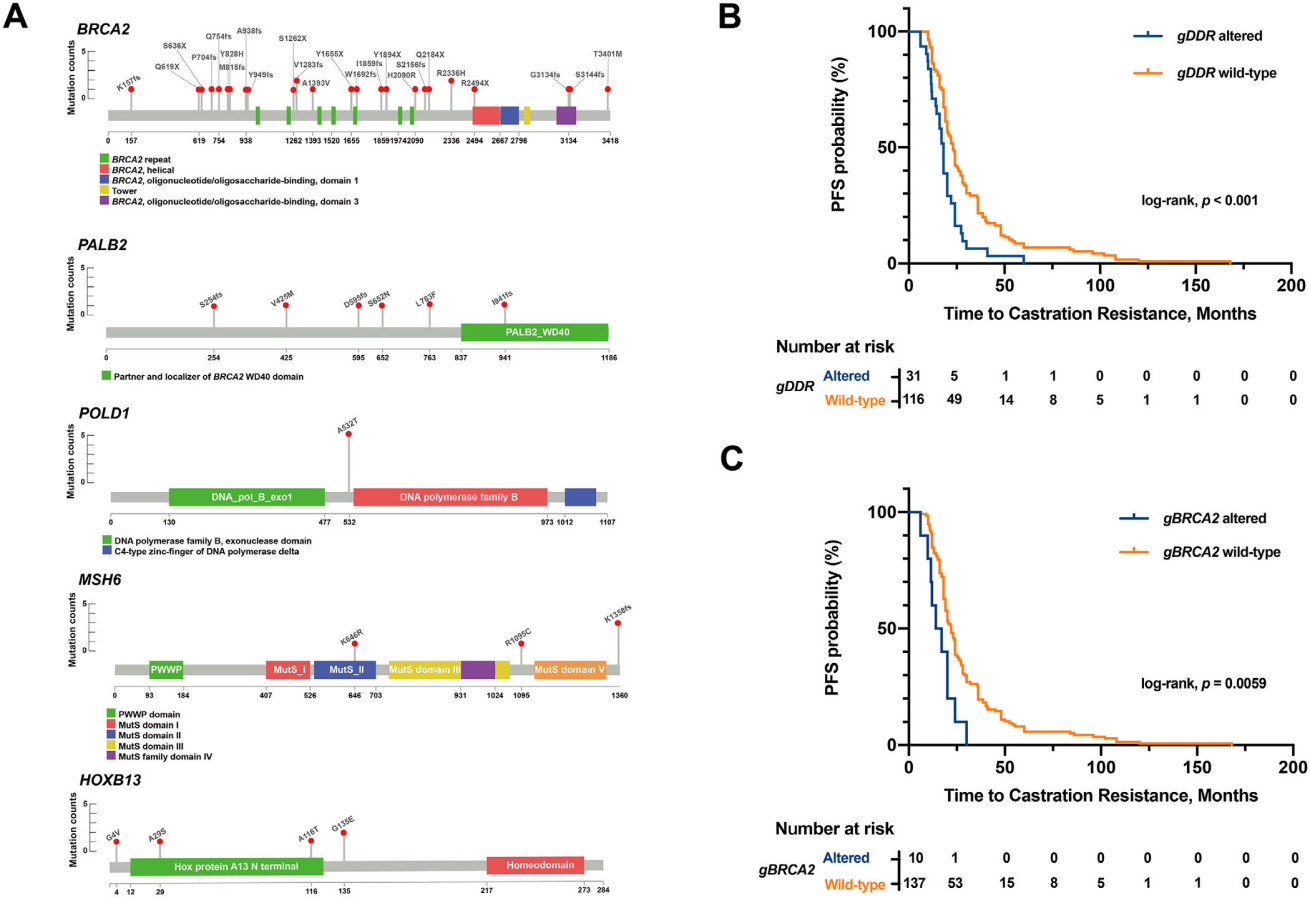
The locations of germline deleterious alterations in the five most frequently altered genes and the association between rare germline deleterious alterations and the time to castration resistance among the patients with metastatic castration‐resistant PCa. (A) Locations of germline deleterious alterations and domains in proteins encoded by the five frequently altered genes are shown by lollipop structures. Protein domains are shown by different colors. On the graph of each gene, the x axis reflects the number of amino acid residues, and the y axis represents the total number of identified germline deleterious alterations. (B) Kaplan–Meier curves for time to castration resistance in patients with germline DDR alteration and germline DDR wild‐type. (C) Kaplan–Meier curves for time to castration resistance in patients with germline *BRCA2* alteration and germline *BRCA2* wild‐type

Additionally, concurrent *HSD3B1* genotype was detected in 348 (71.0%) of the 490 studied patients. To our knowledge, it was the first time to report the genotype of *HSD3B1* in the Chinese population. We compared the alteration frequencies of *HSD3B1* with the cohort by Hearn et al. study,^9^ surprisingly finding a relatively lower incidence of *HSD3B1* c.1245C > A alteration, especially homozygous *HSD3B1* (1245CC) alteration (0.8% in our cohort vs. 7.4% in the cohort by Hearn et al. study, *p *< 0.001) (Figure 1D). *HSD3B1* is responsible for the transformation of steroidal precursors into potent androgens. In addition, *HSD3B1* c.1245A > C was associated with rapid resistance to androgen deprivation therapy but was sensitive to abiraterone.^9^ The rare homozygous alteration of *HSD3B1* in our cohort was also of interest, which might partly interpret the distinct efficacy of conventional hormonal therapy in the Asian population.^2^

Next, we examined the predictive value of germline deleterious alterations in DDR pathway genes. Our results suggested that the germline status of DDR pathway genes was associated with severe disease phenotype and shorter time to castration resistance (18.0 months in the gDDR altered group vs. 23.0 months in the gDDR wild‐type group, *p *< 0.001) (Figure 2B). Specifically, patients harboring deleterious germline *BRCA2* mutation has emerged as a distinct subset with inferior outcomes (15.5 months in the g*BRCA2* altered group vs. 22.0 months in the g*BRCA2* wild‐type group, *p *= 0.0059) (Figure 2C). Nevertheless, recent evidence suggested that those patients harboring germline DDR defect could experience superior clinical outcomes from poly (ADP‐ribose) polymerase inhibitors or platinum‐combined chemotherapy.^1^, ^10^ Thus, we inferred that the patients with metastatic PCa harboring germline deleterious alterations in DDR pathway genes might benefit more from intensive combination therapy, instead of androgen deprivation therapy alone.

In conclusion, we investigated the genomic landscape of rare germline alterations in the Chinese population and highlighted the prognostic value of germline DDR status in general practice. Comparative analysis of the genomic data from our cohort and Caucasian cohorts revealed the interracial diversity in genetic background, suggesting that *PALB2* might be an underlying genomic signature in Chinese population. Especially, the frequency and unique pattern of HOXB13 p.G135E and *HSD3B1* c.1245A > C were unique in the Chinese population. In brief, further investigations by incorporating the genetic background might be helpful to understand the racial diversity and establish therapeutic interventions.

## Supporting information

Supporting information
