# Supplementary material for: The prevalence and clinical implication of rare germline deleterious alterations in Chinese patients with prostate cancer: A real‐world multicenter study
Source: Clin Transl Med. 2021 Oct 12;11(10):e527. doi: 10.1002/ctm2.527 (PMC8506635; doi:10.1002/ctm2.527)
Supplement: Supplementary file 1 — Supporting information [file CTM2-11-e527-s001.docx]

**Supplementary Methods**

1. **Targeted Gene Sequencing**

The NGS test of all samples were performed at GloriousMed Clinical Laboratory (Shanghai) Co., Ltd. For peripheral blood samples, white blood cells (WBCs) were isolated by centrifugation at 1600 × *g* for 10 minutes, and then at 16000 × *g* for 10 minutes. Blood Genomic DNA Mini Kit (Cwbiotech, Beijing, China) was used to extract Genomic DNA (gDNA) from WBCs. 500 ng of gDNA was then used for library preparation and quantification, guided by the protocols of the KAPA Hyper Prep kit (Roche, Basel, Switzerland). Final libraries were sequenced on Illumina Nextseq500 (PE 75) or Novoseq 6000 (PE 150) sequencers (Illumina, San Diego, CA, USA).

1. **Quality control and variant calling**

Trimmomatic [1] was used to trim the sequencing adapters from the raw data. The reads were aligned with the human reference genome (hg19) using BWA [2]. Duplicated reads were removed using Picard (<http://broadinstitute.github.io/picard/>). Mapped reads were realigned to the genome using Genome Analysis Tool Kit ^[3]^. Germline mutations were called using GATK’s Haplotype Caller [3] with a paired workflow. Variants were then annotated using ANNOVAR [4] and in-house developed code. An in-house script was used to verify the human identity concordance of paired samples.

1. **Germline variant filtering**

With a threshold of minimum coverage of 50× and an allele frequency of over 30%, germline variants called by GATK on WBC samples were filtered initially. Then, variants not on coding regions and synonymous mutations annotated using ANNOVAR were filtered out. Furthermore, variants with a population minor allele frequency over 0.1% (annotated using the ExAC database) were considered less functional. Functional filtering removed variants located in non-coding regions and synonymous mutations were removed for downstream analysis. A log2 ratio more than 0.6 was considered a copy gain. A log2 ratio less than −0.7 was considered a copy loss. Deleterious alterations were called when they were nonsense/stop-gains, frameshift insertions and deletions, and ±1, 2 splice-site variants, or were previously reported as pathogenic or likely pathogenic in the ClinVar database.

1. **The measurement time to castration resistance**

The study endpoint was the time from initial hormonal therapy to castration resistance. Castration resistance was defined according to the European Association of Urology (EAU) Guidelines on Prostate Cancer (2021 edition): a. biochemical progression refers to three consecutive rises in prostate specific antigen (PSA) at least one week apart resulting in two 50% increases over the nadir, and a PSA >2 ng/mL; b. radiographic progression refers to the appearance of new lesions including two or more new bone lesions on bone scan or a soft tissue lesion according to Response Evaluation Criteria in Solid Tumors (RECIST). The castrate serum testosterone is defined as <50 ng/dL or 1.7 nmol/L.

1. **Statistical analysis**

All statistical analyses were completed using the R v3.6.0 ([www.R-project.org](http://www.R-project.org)). Clinical characteristics were summarized by different cohorts using descriptive statistics. Fisher’s exact test was used to compare alteration frequencies of individual genes between each cohort. Two-tailed probability value of *p* < 0.05 was considered as statistically significant. The Kaplan-Meier method was used to estimate the time to castration resistance of different groups of patients, and differences between groups were analyzed using the log-rank test in the survival package (v.2.44.1.1). Univariate and multivariate Cox regression analysis were used to calculate the hazard ratios (HR) and 95% confidence intervals (CIs). Only factors significant in univariate analysis were included in the subsequent multivariate analysis.

**References for supplementary methods**

1. Bolger AM, Lohse M, Usadel B: Trimmomatic: a flexible trimmer for Illumina sequence data. *Bioinformatics* 2014, 30(15):2114-20.

2. Li H, Durbin R: Fast and accurate short read alignment with Burrows-Wheeler transform. *Bioinformatics* 2009, 25(14):1754-60.

3. McKenna A, Hanna M, Banks E *et al*: The Genome Analysis Toolkit: a MapReduce framework for analyzing next-generation DNA sequencing data. *Genome Res* 2010, 20(9):1297-303.

4. Wang K, Li M, Hakonarson H: ANNOVAR: functional annotation of genetic variants from high-throughput sequencing data. *Nucleic Acids Res* 2010, 38(16):e164.
